# Validation of the acutely presenting older patient screener for short term mortality prediction in older patients hospitalized for COVID-19

**DOI:** 10.1007/s41999-025-01200-4

**Published:** 2025-04-22

**Authors:** Rosalinde A. L. Smits, Bas F. M. van Raaij, Steffy W. M. Jansen, Jessica M. van der Bol, Carolien M. J. van der Linden, Harmke A. Polinder-Bos, Hanna C. Willems, Ewout W. Steyerberg, Maarten van Smeden, Jacobijn Gussekloo, Simon P. Mooijaart, Stella Trompet

**Affiliations:** 1 Section Gerontology and Geriatrics, Department of Internal Medicine, Leiden University Medical Center, Albinusdreef 2, 2333 ZA Leiden, The Netherlands; 2 Department of Geriatrics, Catharina Hospital, Eindhoven, The Netherlands; 3 Department of Geriatric Medicine, Reinier de Graaf Hospital, Delft, The Netherlands; 4 Division of Geriatrics, Department of Internal Medicine, Erasmus MC, University Medical Center, Rotterdam, The Netherlands; 5 Section Geriatrics, Department of Internal Medicine, Amsterdam University Medical Center, Location AMC, Amsterdam, The Netherlands; 6 Department of Biomedical Data Sciences, Leiden University Medical Centre, Leiden, The Netherlands; 7 Julius Center for Health Sciences and Primary Care, Utrecht University Medical Center, Utrecht, The Netherlands; 8 Department of Public Health and Primary Care, LUMC Center for Medicine for Older People, Leiden University Medical Centre, Leiden, The Netherlands; 9 Section Gerontology and Geriatrics, Department of Internal Medicine, Leiden University Medical Centre, Leiden, The Netherlands; 10 LUMC Center for Medicine for Older People, Leiden University Medical Centre, Leiden, The Netherlands

**Keywords:** Screening instrument, Validation, Older patients, Mortality

## Abstract

**Purpose:**

The aim of this study was to validate the acutely presenting older patient (APOP) screener, routinely used on the Emergency Department to predict risk of adverse outcomes in older people, for prediction of in-hospital mortality and 30-days-mortality in older patients hospitalized for COVID-19.

**Methods:**

Patients ≥ 70 years from a multicenter cohort hospitalized for COVID-19 with measured APOP risk were included. External validation analysis of the APOP screener for in-hospital mortality and 30-days-mortality was performed including discrimination and calibration.

**Results:**

389 patients (median age 80 (IQR 75–85) years, 41.4% female, 138 APOP high risk) were included. APOP high risk patients more often lived institutionalized, (26% vs. 4%; p < 0.001), had more comorbidities (Charlson Comorbidity Index 2 (1–3) vs. 2 (0–3); p = 0.002) and were less often fit (Clinical Frailty Scale 1–3 17% vs. 62%; p < 0.001). 84 patients died in hospital and 114 within 30 days. APOP high risk patients had a higher risk of in-hospital-death [OR 1.6 (95% CI 1.0–2.6)] and death within 30 days [OR 2.7 (95% CI 1.7–4.2)]. The APOP screener discriminated poorly for in-hospital mortality [AUC 0.56 (95% CI 0.48–0.63)] and for 30-days-mortality [AUC 0.62 (95% CI 0.55–0.68)]. Calibration plots revealed overestimation of the screener for both mortality risks.

**Conclusion:**

The APOP screener had a poor predictive performance for in-hospital mortality and 30-days-mortality in older people hospitalized for COVID-19. Screening tools routinely used on the ED may not be useful to predict mortality in different than usual clinical circumstances such as during a pandemic of a novel disease.

**Supplementary Information:**

The online version contains supplementary material available at 10.1007/s41999-025-01200-4.

## Key summary points

**Aim:**

To validate the acutely presenting older patient (APOP) screener to predict risk of adverse outcomes in older people, for prediction of in-hospital mortality and 30-days-mortality in older patients hospitalized for COVID-19.

**Findings:**

The APOP screener discriminated poorly for in-hospital mortality [AUC 0.56 (95% CI 0.48–0.63)] and for 30-days-mortality [AUC 0.62 (95% CI 0.55–0.68)]. Calibration plots revealed overestimation of the screener for both mortality risks.

**Message:**

Screening tools routinely used on the ED may not be useful to predict mortality in different than usual clinical circumstances such as during a pandemic of a novel disease.

**Supplementary Information:**

The online version contains supplementary material available at 10.1007/s41999-025-01200-4.

## Introduction

During the COVID-19 pandemic, health care professionals working on the Emergency Department (ED) needed to assess appropriate treatment for large groups of acutely ill COVID-19 patients, due to pressure on hospital admission capacity. Older patients, especially those living with frailty, had a high risk of dying of COVID-19 in hospital [1]. Several screening instruments are available to predict individual risk for adverse outcomes in older people, based on their level of frailty. In many Dutch hospitals the Acute Presenting Older Patient (APOP) screener, predicting the risk of functional decline and mortality in older people, is routinely used on the ED [2]. The APOP screener has been developed prior to the COVID-19 pandemic [3], but can potentially be used to predict individual risk of mortality during a pandemic.

Therefore, the aim of this study was to validate the APOP screener for prediction of in-hospital mortality and 30-days-mortality in older patients hospitalized for COVID-19.

## Methods

### Study design and setting

The COVID-OLD is a multicenter cohort study among patients aged ≥ 70 years hospitalized for COVID-19 between February 2020 and April 2022 in The Netherlands. Data were collected from 5 Dutch hospitals where the APOP screener has been implemented to routine ED care: Catharina Hospital (Eindhoven), Deventer Hospital (Deventer), Reinier de Graaf Hospital (Delft), Sint Jansdal Hospital (Harderwijk) and Leiden University Medical Centre (Leiden). Further details of the study design can be found in an earlier publication [4].

### Study participants

Patients were eligible for inclusion in the COVID-OLD cohort if they were ≥ 70 years, admitted for COVID-19 and not transferred between hospitals, because of incomplete data of these patients. Diagnosis of COVID-19 was confirmed with a positive polymerase chain reaction (PCR) test-result from a nasal or oropharyngeal swab or based on symptoms, radiological abnormalities and laboratory findings in the first pandemic wave in 2020. Furthermore, patients with unknown or incomplete APOP score were excluded.

### Acutely presenting older patient (APOP) screener

The APOP screener is a prediction instrument for the risk of mortality and/or functional decline 3 months after ED presentation [2]. The screener is applied on the ED during triage by a nurse and takes only a few minutes. The screener consists of nine questions concerning age, sex, arrival by ambulance, need for help on a regular basis, need for assistance in bathing or showering, hospitalization in the last 6 months, dementia diagnosis and, in case there is no dementia diagnosis, 2 questions to test cognition; the patient should name the current year and the months of the year in reversed order. These 9 items build up to the individual risk for mortality and/or functional decline. The screener shows two outcomes: first, an individual composite outcome, expressed as a percentage that indicates the individual risk of functional decline and/or death within the next 3 months (component 1) and second, the presence of signs of impaired cognition as a yes or no outcome (component 2). The cut-off value indicating a high risk of functional decline and/or death is ≥ 45% [3]. In most hospitals the screener has been integrated in the electronic health record. The APOP screener has also been developed to be used in selecting high risk hospitalized patients for geriatric consultation and a comprehensive geriatric assessment during admission.

### Clinical frailty scale (CFS)

The Clinical Frailty Scale is a 9-point scale of the level of frailty and includes physical fitness and dependency on others for daily activities. Originally it was designed not only to measure frailty but also to predict death or the need for an institution [5]. The patient is asked about mobility and need for help in daily life two weeks before the acute illness occurred. In our study frailty is categorized in two groups: fit (CFS 1–3) and frail (CFS 4–9). The CFS is internationally recommended to be used on the ED for frailty assessment of older patients with COVID-19 [6].

### Data collection

Data were collected from the patient’s electronic healthcare records. We collected demographic data on age, sex and living situation (at home or institutionalized). The Charlson Comorbidity Index (CCI) was used to assess the presence of comorbidities [7]. Physical impairment was evaluated using the Katz Activities of Daily Living (ADL) Index [8]. A score ≥ 2 is defined as risk of physical impairment. The APOP score was prospectively determined at the ED and defined as low (< 45%) and high ≥ 45%) risk score. The presence of signs of impaired cognition adds to a higher individual risk and has not been analyzed separately. The CFS was preferably prospectively determined at hospital admission. If not prospectively assigned, the CFS was determined retrospectively, based on available chart data (which included the geriatric parameters from the VMS; Safety Management System, a national in hospital screening instruments for older patients [9]) and was scored by a researcher trained by a geriatrician or internist-geriatrician. Mortality was scored at discharge (in-hospital mortality) and 30 days after admission (30-days-mortality).

Data were collected using Castor Electronic Data Capture (2022).

### Statistical analyses

External validation of the APOP screener for in-hospital and 30-days-mortality was performed in 4 steps. First, we compared baseline clinical characteristics between the low risk and high-risk groups according to the APOP screener. Baseline characteristics were analyzed with descriptive statistics. Continuous data are presented with the mean and standard deviation (SD), or median and interquartile range (IQR) based on the normality of distribution. Categorical data are presented with absolute values and percentages. Second, the association of the APOP screener with in-hospital and 30-days-mortality was assessed with binary logistic regression. Third, discrimination of the APOP screener was assessed with the area under the curve (AUC) of the receiver operating characteristic (ROC) curve. An AUC of 0.5 is equal to chance, while 1 is perfect discrimination [10]. Accuracy of the screener was further assessed with sensitivity and specificity. Fourth, calibration was visually assessed with calibration curves of the observed risk versus the predicted risk using the APOP screener. The ratio of observed to expected risk was calculated as a summary measure for calibration.

To compare predictive performance of the APOP screener with another routinely used screener, we assessed the association with and discrimination of CFS with in-hospital and 30-days-mortality. We also assessed the association with and discrimination of age to evaluate whether age alone accounts for most of the predictive performance for mortality. Agreement between APOP and CFS was measured using kappa, where 0.80 and higher means a good agreement.

The reported p-values are two-sided and p-values < 0.05 are considered statistically significant unless otherwise specified. Statistical analysis was performed with R, version 4.3.1 and IBM SPSS Statistics, version 25.

## Results

### Characteristics of study participants

**Fig. 1:**
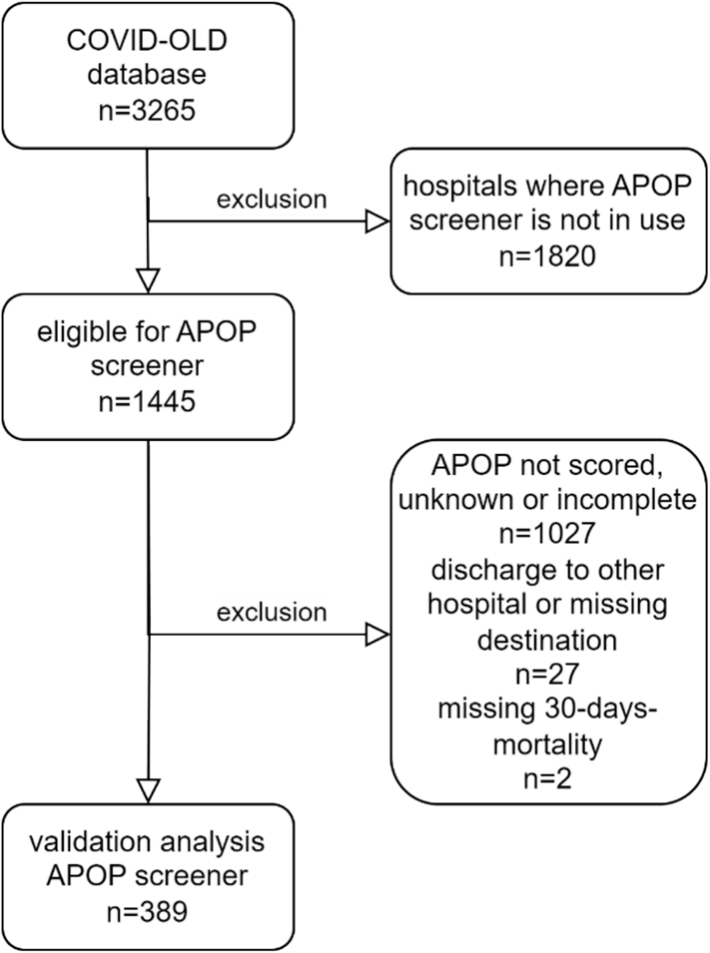
Flowchart

A total of 1445 patients were eligible for participation (Fig. 1). We excluded 1027 patients because the APOP screener was not (fully) scored, 27 patients were discharged to another hospital or unknown destination and for 2 patients 30-days-mortality was missing, resulting in 389 patients included for validation. Included and not-included patients differed with respect to percentage of patients living institutionalized (included 11.9% vs not-included 8.3%; p = 0.035), history of dementia (included 11.0% vs. not-included 7.4%; p = 0.026) and division into CFS groups (fit group (CFS 1–3) included 48.4% vs. not-included 37.4%; p = 0.002) (supplemental Table 1).

A total of 251 APOP low risk (39.4% female) and 138 APOP high risk patients (44.9%) were included (Table 1). Compared to patients with a low risk and inherent to the elements of the screener itself, APOP high risk patients had a higher median age [85 years (IQR 80–89) vs. 77 years (IQR 74–82)], more often needed assistance in personal care [Katz ADL score 2 (IQR 0–5) vs. 0 (IQR 0–1)] and more often have been diagnosed with dementia (25% vs. 5%). Furthermore, APOP high risk patients more often lived institutionalized (26% vs. 4%; p < 0.001), had more comorbidities [CCI 2 (1–3) vs. 2 (0–3); p 0.002] and were less often fit (CFS 1–3 17% vs. 62%; p < 0.001). APOP high risk patients presented themselves earlier in the course of the disease [4 days (IQR 1–7) vs. 6 days (IQR 3–9); p = 0.005] and with lower CRP levels at admission [55 (24–102) vs. 71 (30–122); p 0.059]. Other disease severity indicators at time of admission (temperature, respiratory rate, oxygen amount) were similar in both groups.

**Table 1:** Baseline characteristics for older hospitalized COVID-19 patients at time of admission categorized by APOP risk

	APOP low risk	APOP high risk*	p-Value
	N = 251	N = 138	
Demographics			
Age (years), median (IQR)	77 (74–82)	85 (80–89)	NA
Female sex, n (%)	99 (39.4)	62 (44.9)	NA
Living institutionalized, n (%)	11 (4.4)	36 (26.3)	< 0.001
Comorbidity			
Charlson Comorbidity Index, median (IQR)	2 (0–3)	2 (1–3)	0.002
History of dementia, n (%)	12 (4.8)	34 (24.6)	NA
Geriatric measurements			
Katz ADL score, median (IQR)	0 (0–1)	2 (0–5)	NA
Use of walking aid, n (%)	62 (28.3)	90 (75.6)	< 0.001
Clinical Frailty Scale			
CFS per group			< 0.001
1–3 (fit)	141 (61.8)	20 (16.8)	
4–5 (pre-frail)	55 (24.1)	27 (22.7)	
6–9 (frail)	32 (12.7)	72 (60.5)	
Disease severity indicators			
Duration of symptoms until admission (days), median (IQR)	6 (3–9)	4 (1–7)	0.005
Body temperature (°C), mean (SD)	37.8 (1.2)	37.6 (1.1)	0.168
Respiratory rate (breaths/min), median (IQR)	20 (17–25)	20 (16–25)	0.785
Oxygen amount needed (L/min), median (IQR)	2 (0–4)	2 (0–4)	0.786
C-reactive protein (mg/L), median (IQR)	71 (30–122)	55 (24–102)	0.059

Analysis: Chi square test/Mann Whitney U test, Missing total group: 3 living institutionalized, 48 Katz ADL score, 51 use of walking aid, 42 Clinical Frailty Scale, 30 duration of symptoms, 8 body temperature, 18 respiratory rate, 25 oxygen amount needed, 8 C-reactive protein, Missing APOP low risk: 2 living institutionalized, 26 Katz ADL score, 32 use of walking aid, 23 Clinical Frailty Scale, 18 duration of symptoms, 7 body temperature, 15 respiratory rate, 15 oxygen amount needed, 5 C-reactive protein, Missing APOP high risk: 1 living institutionalized, 22 Katz ADL score, 19 use of walking aid, 19 Clinical Frailty Scale, 12 duration of symptoms, 1 body temperature, 3 respiratory rate, 10 oxygen amount needed, 3 C-reactive protein

*N * number, *IQR* interquartile range, *SD* standard deviation, *SE* standard error, *ADL* activities of daily living

*45% and more risk for mortality or functional decline in the coming 3 months on APOP screener

### Main results

In total 84 (21.6%) patients died in hospital and 114 (29.3%) died within 30 days. APOP high risk patients had a higher risk of death in hospital [OR 1.6 (95% CI 1.0–2.6); p = 0.065] and death within 30 days [OR 2.7 (95% CI 1.7–4.2); p < 0.001] (Table 2). The risk of in-hospital mortality was also higher with CFS ≥ 4 [OR 1.9 (95% CI 1.1–3.3); p = 0.018]. The risk of mortality within 30 days is also higher with CFS ≥ 4 [OR 2.9 (95% CI 1.7–4.8); p < 0.001] and with age [OR 1.1 (95% CI 1.0–1.1) per year; p < 0.001].

**Table 2:** Association of screening instruments with mortality of older hospitalized COVID-19 patients

In-hospital mortality				30-days-mortality		
Variable	n = /N =	OR (95% CI)	p-value	n = /N =	OR (95% CI)	p-value
APOP						
Low risk	47/251	Ref		55/251	Ref	
High risk^a^	37/138	1.59 (0.97–2.60)	0.065	59/138	2.66 (1.70–4.18)	< 0.001
CFS						
Low	24/161	Ref		28/161	Ref	
High^b^	47/186	1.93 (1.12–3.30)	0.018	70/186	2.87 (1.73–4.75)	< 0.001
Age^c^	84/389	1.02 (0.99–1.06)	0.220	114/389	1.06 (1.03–1.10)	< 0.001

*n* number of deceased patients, *N* total number of cases without missing values, *Analysis* binary univariate logistic regression analysis, *Missing* 42 CFS, *N* number

^a^45% and more risk for mortality or functional decline on APOP screener

^b^CFS ≥ 4

^c^Continuous per year

The APOP screener differentiated poorly between death and alive during hospital stay with an AUC of 0.56 (95% CI 0.48–0.63) and between death and alive within 30 days after admission with an AUC of 0.62 (95% CI 0.55–0.68) (Table 3). CFS differentiated poorly between death and alive during hospital stay with an AUC of 0.58 (95% CI 0.51–0.65) and between death and alive within 30 days with an AUC of 0.62 (95% CI 0.56–0.69). Age differentiated poorly between death and alive during hospital stay with an AUC of 0.54 (95% CI 0.46–0.61) and between death and alive within 30 days with an AUC of 0.59 (95% CI 0.53–0.65). The correlation between APOP and CFS had a kappa of 0.40, which is rather low.

**Table 3:** Discrimination of screening instruments for mortality of older hospitalized COVID-19 patients

Variable	In-hospital mortality			30-Days-mortality		
	AUC	Sensitivity	Specificity	AUC	Sensitivity	Specificity
APOP high risk^a^	0.56 (0.48–0.63)	44 (33–55)	67 (61–72)	0.62 (0.55–0.68)	52 (42–61)	71 (66–77)
High CFS^b^	0.58 (0.51–0.65)	66 (54–77)	50 (44–56)	0.62 (0.56–0.69)	71 (61–80)	53 (47–60)
High age^c^	0.54 (0.46–0.61)	52 (41–63)	55 (50–61)	0.59 (0.53–0.65)	59 (49–68)	59 (53–65)

Analysis sensitivity/specificity Clopper-Pearson CI. All values between brackets are 95% confidential intervals. All numbers > 1 are percentages

^a^45% and more risk for mortality or functional decline on APOP screener

^b^CFS ≥ 4

^c^ > 80 years

**Fig. 2:**
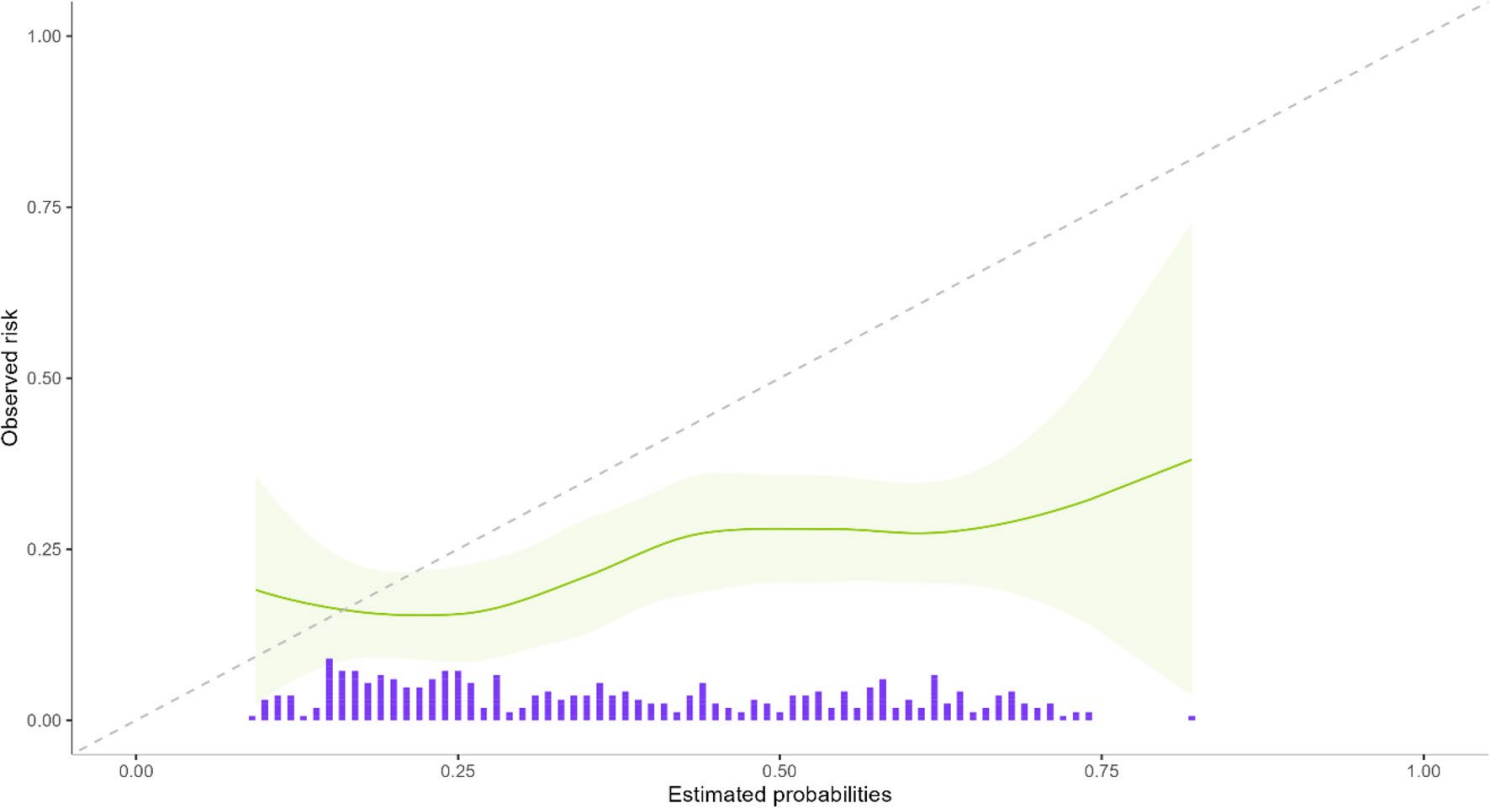
Calibration curve in-hospital mortality (green line: observed versus estimated risk green band: range purple bars: relative numbers of patients per risk)

**Fig. 3:**
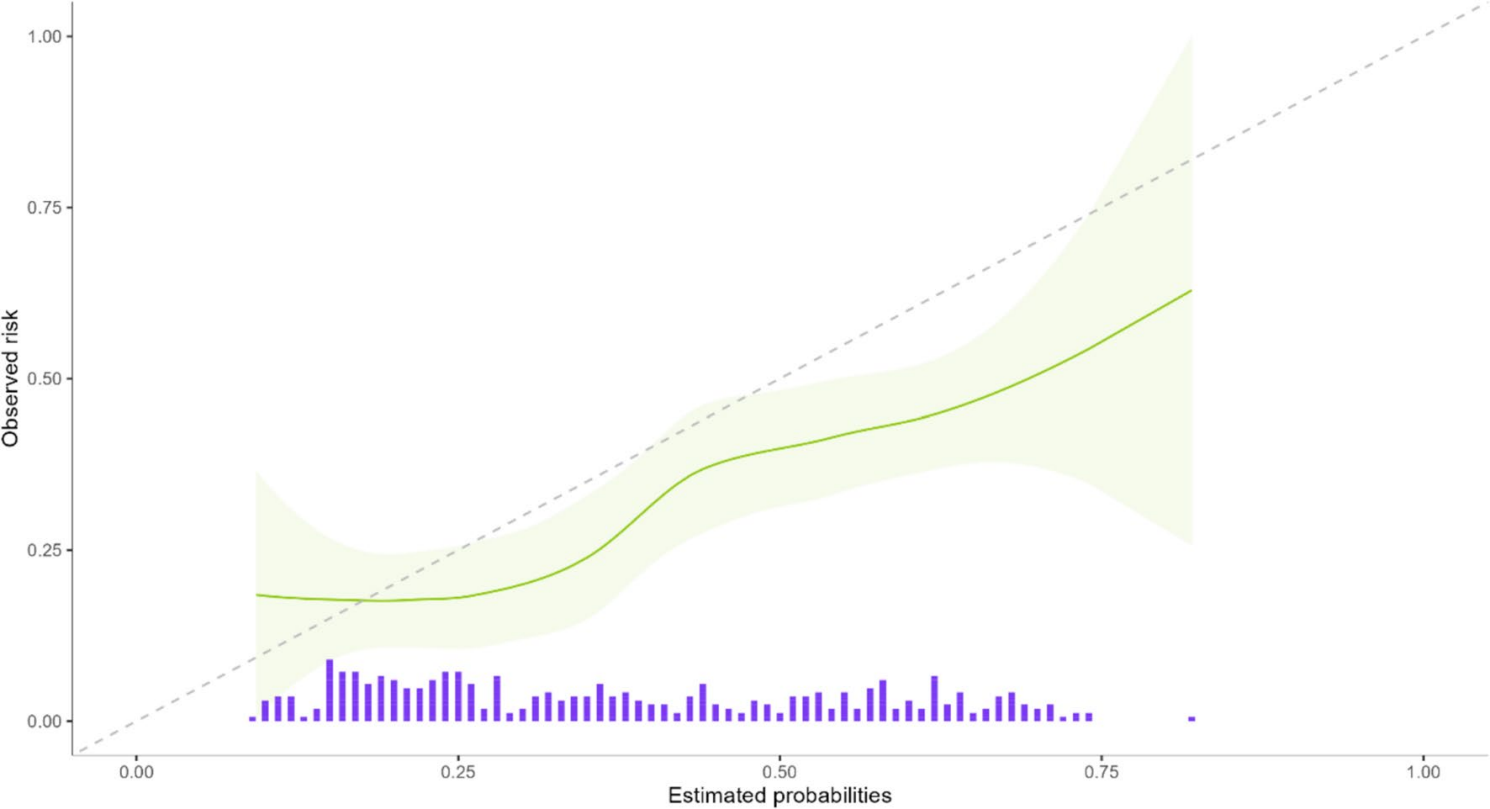
Calibration curve 30-days-mortality (green line: observed versus estimated risk green band: range purple bars: relative numbers of patients per risk)

The calibration plots of the APOP screener showed an overestimation of observed outcomes versus predicted outcomes (Figs. 2 and 3). The vertical bars represent the number of patients per estimated probability. For in-hospital mortality the calibration slope was 0.33 (95% CI 0.04–0.62), for 30-days-mortality the calibration slope was 0.65 (95% 0.38–0.92).

## Discussion

Validation of the APOP screener demonstrated a poor predictive performance in terms of discrimination and calibration for both in-hospital mortality and 30-days-mortality in older patients hospitalized for COVID-19. The discriminative performance of the CFS and age was equally poor compared to the performance of the APOP screener.

The APOP screener has been developed and validated in cohorts of patients on the ED with an acute disease prior to the start of the COVID-19 pandemic. The APOP development and validation study [3] was performed in four hospitals in patients with acute presentations on the ED. They showed that the APOP screener differentiated fairly between alive after 90 days without functional decline and death within 90 days or functional decline with an AUC of 0.74. Patient characteristics were comparable to the characteristics in the present study [3]. A single center validation study of the APOP screener in older COVID-19 patients on the ED [11] demonstrated a poor predictive performance comparable to the performance in the present study. They demonstrated that the APOP screener differentiated between death and alive during hospital stay with an AUC of 0.59. Patients were less fit compared to the patients in the present study (CFS 1–3 34.6% vs. 48.4%) [11]. Other prediction models for older patients specifically developed during the COVID-19 pandemic are the Risk Stratification in the Emergency Department in Acutely Ill Older Patients (RISE UP) including age, vital signs and laboratory parameters and the 4C mortality score including age, comorbidity, vital signs and laboratory parameters. Both do not include parameters related to frailty or cognition. A validation study of the RISE UP and 4C mortality score [12] included older COVID-19 patients (median age 71 years) presenting to the ED with COVID-19 in the first pandemic wave. The RISE UP differentiated good between death and alive within 30 days after admission with an AUC of 0.83 [12], similar to the predictive performance of the RISE UP for patients with other acute diseases [13]. In the study of van Dam et al., the 4C mortality score differentiated good between death and alive within 30 days after admission with an AUC of 0.84 [12]. A second study of older COVID-19 patients in the COVID-OLD cohort (Zahra et al. 2024) showed a fair discriminative performance for in-hospital mortality with an AUC 0.74 (0.72–0.76) for 4C mortality score; discrimination in two other COVID-19 cohorts was lower (0.66 and 0.70) [14]. In conclusion, the APOP screener did not perform well in predicting mortality in COVID-19 patients compared to predicting mortality in patients with other acute diseases and did not perform well in predicting mortality in COVID-19 patients compared to other prediction models.

There are at least three possible explanations for the poor performance of the APOP screener in predicting short term mortality in older COVID-19 patients on the ED in this present study. First, the APOP screener only covers a selection of variables per patient, reflecting frailty. The predictors of the APOP screener do not reflect severity of acute illness, although severe illness could significantly influence individual prognosis. This is supported by the finding that CFS and age have a similar poor predictive performance for mortality prediction among older COVID-19 patients. Furthermore, the RISE UP and 4C mortality perform much better in predictions than the APOP screener. These models include parameters for severity of illness (vital signs and laboratory parameters). Therefore, it is possible that in the case of COVID-19, frailty has little impact on the risk of dying compared to the impact of severity of illness. Second, the poor performance of the APOP screener could be explained by the dynamic context of the COVID-19 pandemic. Mortality rates changed in relatively short time frames due to improved treatment and prevention options or differences in disease characteristics caused by viral mutations. During the COVID-19 pandemic, mortality rates were the highest early 2020, when nobody had immunity for the virus and treatment options were limited [4]. After the introduction of vaccination (early 2021), mortality rates of older patients decreased [15]. The patients included in this present study were admitted during the entire two-year period of the pandemic in the Netherlands and 80 patients (20.6%) were vaccinated. Therefore, the APOP screener could have a different predictive performance during the first stage of the pandemic or among vaccinated patients only. The small sample size of this study hampers additional analysis to evaluate whether the predictive performance changed due to pandemic stage or vaccinations.

Third, there might have been selection bias, because many patients were not included because the APOP screener was not complete, and a relatively large number of included patients were fit and therefore had low risk score with the APOP screener.

Compared to the APOP screener, models that include acute illness parameters (RISE UP and 4C mortality score) have a better predictive performance for mortality in older COVID-19 patients on the ED. The internationally recommended CFS is also outperformed by the predictions of models that include acute illness parameters, shown by comparing the AUC of the CFS in this study to the AUC’s of the RISE UP and 4C mortality score in previous studies as described earlier in the discussion. Therefore, in future cases of novel (viral) disease pandemics, it may be better to use models that include acute illness parameters for mortality predictions in older people and to validate these models in the new population before implementing them in guidelines. The RISE UP and 4C mortality score show reliable predictive performance in earlier COVID-19 studies and can be a good choice. Recently, a new prediction model has been developed in the COVID-OLD cohort including age, comorbidity, CFS, vital signs and laboratory parameters predicting in-hospital mortality. The model has been developed for patients admitted in the first pandemic wave and showed a good discriminative performance with an AUC of 0.80 (95% CI 0.76–0.84) [16]. Because mortality is not the only important outcome for older patients, in future studies it may also be useful to validate the APOP screener and CFS for more patient-centered outcomes, for example functional decline or quality of life.

This study has some weaknesses. Originally the APOP screener has been developed and validated for 90-days-mortality and functional decline, these outcomes were not collected in the COVID-OLD cohort. In the COVID-OLD cohort only in-hospital mortality and 30-days-mortality have been collected. However, for clinical implications, in-hospital and 30-days-mortality are more relevant for decision making concerning extensive therapy (e.g., ICU admission)) and advance care planning towards rehabilitation or end-of-life-care. The total study population was relatively small compared to the total group of patients that could have been eligible for APOP screener use. However, because baseline characteristics of included and not included patients are not clinically different, we opine that the selected group is representative for the older population in need of hospitalization for COVID-19. One strength of the present study was that screening included patients with the APOP screener followed routine clinical care, increasing the relevance for daily practice of the outcomes of the present study. Another strength of the present study is that we used step-by-step validation of in-hospital and 30-days-mortality in a multicenter cohort of the APOP screener including discrimination, calibration, and a comparison to another well-used screening instrument. In this way we intended to perform a reliable and clinically relevant predictive performance analysis.

Taken together, the APOP screener had a poor predictive performance for in-hospital mortality and 30-days-mortality in older people hospitalized for COVID-19. This implies that screening tools routinely used on the ED may not be useful to predict mortality in different than usual clinical circumstances such as during a pandemic of a novel disease.

## Supplementary Information

Below is the link to the electronic supplementary material.

Supplementary file1 (DOCX 16 KB)

## Data Availability

The data that support the findings of this study are available from the corresponding author, upon reasonable request.
