# Supplementary material for: Validation of the acutely presenting older patient screener for short term mortality prediction in older patients hospitalized for COVID-19
Source: Eur Geriatr Med. 2025 Apr 22;16(4):1519–26. doi: 10.1007/s41999-025-01200-4 (PMC12378732; doi:10.1007/s41999-025-01200-4)
Supplement: Supplementary file 1 — Supplementary file1 (DOCX 16 KB) [file 41999_2025_1200_MOESM1_ESM.docx]

| **Supplemental table 1.** Baseline characteristics for older hospitalized COVID-19 patients eligible for APOP screening | | | | |
| --- | --- | --- | --- | --- |
|  | **total group** | **APOP complete** | **APOP missing/incomplete** | **p-value** |
|  | **N= 1445** | **N= 418** | **N= 1027** |  |
| **Demographics** |  |  |  |  |
| Age (years), median (IQR) | 79 (74-84) | 80 (75-85) | 79 (74-84) | 0.056 |
| Male sex, n (%) | 877 (60.8) | 248 (59.3) | 629 (61.4) | 0.493 |
| Living institutionalized, n (%) | 131 (9.3) | 49 (11.9) | 82 (8.3) | 0.035 |
| **Comorbidity** |  |  |  |  |
| Charlson Comorbidity Index, median (IQR) | 2 (1-3) | 2 (1-3) | 2(1-3) | 0.781 |
| History of dementia, n (%) | 122 (8.4) | 46 (11.0) | 76 (7.4) | 0.026 |
| **Geriatric measurements** |  |  |  |  |
| Katz ADL score, median (IQR) | 0 (0-2) | 0 (0-2) | 0 (0-2) | 0.277 |
| Use of walking aid, n (%) | 490 (41.0) | 158 (43.8) | 332 (39.8) | 0.184 |
| CFS per group, n (%) |  |  |  | 0.002 |
| 1-3 (fit) | 457 (41.1) | 180 (48.4) | 277 (37.4) |  |
| 4-5 (pre-frail) | 298 (26.8) | 84 (22.6) | 214 (28.9) |  |
| 6-9 (frail) | 358 (32.2) | 108 (29.0) | 250 (33.7) |  |
| **Disease severity indicators** |  |  |  |  |
| Duration of symptoms until admission (days), median (IQR) | 6 (3-10) | 6 (2-9) | 6 (3-10) | 0.065 |
| Body temperature (°C), mean (SD) | 37.6 (1.15) | 37.7 (1.2) | 37.6 (1.1) | 0.185 |
| Respiratory rate (breaths/min), median (IQR) | 20 (18-25) | 20 (17-25) | 21 (18-25) | 0.227 |
| Oxygen amount needed (L/min), median (IQR) | 2 (0-4) | 2 (0-4) | 2 (0-4) | 0.585 |
| C-reactive protein (mg/L), median (IQR) | 70 (34-130) | 66 (29-120) | 71 (35-130) | 0.094 |
| Abbreviations: N=number; IQR=interquartile range; SD=standard deviation; SE=standard error; ADL=Activities of Daily Living  Analysis: independent T test / Chi square test / Mann Whitney U test  Missing total group: 2 sex, 39 living institutionalized , 262 Katz ADL score, 250 use of walking aid 332 Clinical Frailty Scale, 139 duration of symptoms, 35 body temperature, 72 respiratory rate, 100 oxygen amount needed, 31 C-reactive protein  Missing APOP scored: living institutionalized, 54 Katz ADL score, 57 use of walking aid 46 Clinical Frailty Scale, 30 duration of symptoms, 8 body temperature, 19 respiratory rate, 26 oxygen amount needed, 8 C-reactive protein  Missing APOP not scored: 2 sex, living institutionalized , 208 Katz ADL score, 193 use of walking aid 286 Clinical Frailty Scale, 109 duration of symptoms, 27 body temperature, 53 respiratory rate, 74 oxygen amount needed, 23 C-reactive protein | | | | |
